# Peritoneal dialysis influences microRNA expression and pro-inflammatory response: results from a cross-sectional study

**DOI:** 10.1590/2175-8239-JBN-2024-0218en

**Published:** 2025-12-19

**Authors:** Nara Aline Costa, Amanda Gomes Pereira, Hellen Christina Neves Rodrigues, Lilian Cuppari, Tainara Francini Felix, Iael Weissberg Minutentag, Patricia Pintor Reis, André Luis Balbi, Bertha Furlan Polegato, Paula Schmidt Azevedo, Leonardo Antonio Mamede Zornoff, Sérgio Alberto Rupp de Paiva, Daniela Ponce, Marcelo Macedo Rogero, Marcos Ferreira Minicucci

**Affiliations:** 1 Universidade Federal de Goiás, Faculdade de Nutrição, Goiânia, GO, Brazil.; 2 Universidade Estadual Paulista, Faculdade de Medicina, Departamento de Medicina Interna, Botucatu, SP, Brazil.; 3 Universidade Federal de São Paulo, Disciplina de Nefrologia, São Paulo, SP, Brazil.; 4 Universidade Estadual Paulista, Faculdade de Medicina, Unidade de Pesquisa Experimental, Botucatu, SP, Brazil.; 5 Universidade Estadual Paulista, Faculdade de Medicina, Departamento de Cirurgia e Ortopedia, Botucatu, SP, Brazil.; 6 Universidade de São Paulo, Faculdade de Saúde Pública, Departamento de Nutrição, São Paulo, SP, Brazil.

**Keywords:** Renal Insufficiency, Chronic, MicroRNAs, Inflammation, Metabolic Networks and Pathways

## Abstract

**Introduction:**

The investigation of circulating microRNAs (miRNAs) and inflammatory response associated with the different stages of chronic kidney disease (CKD) may reveal biomarkers of disease pathogenesis. Our goal was to identifying differences in the circulating miRNAs expression between peritoneal dialysis (PD) and non-dialytic (ND) patients and determine the regulatory miRNA-target gene networks and pathways potentially involved in disease pathogenesis.

**Methods:**

This was an exploratory cross-sectional study that included ND and PD patients with CKD stage 5 over 18 years of age. Inflammatory biomarkers and circulating miRNA expression profiles were evaluated.

**Results:**

The study included 20 patients (57.2 ± 11.8 years). Levels of high-sensitivity C-reactive protein [0.37 (0.07–1.40) vs. 3.90 (2.50–5.79), p = 0.038] and interleukin-6 [3.35 ± 3.08 vs. 6.82 ± 4.08, p = 0.046] were significantly lower in the ND group in comparison to the PD group, respectively. Nine miRNAs were significantly deregulated (fold change (FC) ≥ 2 and p ≤ 0.05) in the PD compared to the ND group. Computational analyses showed a large number of target genes commonly regulated by at least two of the identified miRNAs. Pathway enrichment analysis showed that G protein-coupled receptor (GPCR) signaling, insulin secretion/resistance, and energy metabolism were among significant pathways regulated by miRNA target genes.

**Conclusions:**

Patients on PD treatment showed deregulated circulating levels of the 9 identified miRNAs and higher serum inflammatory biomarkers, compared to ND patients. Genes regulated by miRNAs are mainly associated with GPCR signaling, insulin resistance, and energy metabolism, playing roles in fibrosis and inflammatory-associated functions.

## Introduction

Chronic kidney disease (CKD) stages 1–5 affects over 10% of the world population—equivalent to 843.6 million individuals. The burden of CKD represents a significant global health challenge, especially in low- and middle-income countries, as it has become a leading cause of mortality, highlighting the urgent need for improved prevention and treatment efforts^1^. Progression to end stage CKD requires treatment with renal replacement therapies, including peritoneal dialysis (PD). Patients on non-dialysis (ND) treatment typically exhibit a lower pro-inflammatory state due to the preservation of residual renal function, which allows for the clearance of inflammatory cytokines. In contrast, individuals undergoing PD often present with increased inflammation, attributed to factors such as insulin resistance, hyperglycemia, weight gain, peritonitis, and catheter-related infections^2,3^. Furthermore, it is known that adipose tissue and greater inflammation intensity contribute to increased morbidity and mortality, especially among patients on dialysis therapy^4,5,6,7^. These differences highlight the importance of delaying CKD progression to dialysis stages, as maintaining renal function is crucial for mitigating inflammation and disease progression.

In this perspective, new lines of research have suggested that microRNAs (miRNAs), associated with systemic features such as vascular calcification, atherosclerosis, and mineral and bone disorders, play a role in the development and progression of CKD^8^. miRNAs are small, non-coding RNAs with a post-transcriptional regulatory role in gene expression^9,10^. Minimally invasive strategies have been developed to quantify miRNA levels in body fluids, such as blood, from patients with numerous clinical conditions. Analysis of plasma miRNAs offers numerous advantages, since these molecules are abundant in body fluids, have high stability, and are easy to detect^11^. Of note, miRNAs regulate target genes involved in important biological processes, including fibrosis in response to tissue injury and renal damage^8^. Furthermore, miRNAs, including miR-155, miR-146a and miR-21, have been implicated in the regulation of inflammatory response, and if altered, can contribute to intense depletion of muscle mass^12,13^.

Studies suggest that there is a complex and integrated relationship between miRNAs, immune and inflammatory responses, and body composition, but published data are insufficient for the complete understanding of this relationship in CKD^14,15^. To the best of our knowledge, no study has assessed the levels of circulating miRNAs in non-dialytic (ND) and PD patients. We aimed to identify differences in the expression of circulating miRNAs between PD and ND patients and determine the regulatory miRNA-target gene networks and pathways potentially involved in disease pathogenesis.

## Methods

### Study Design and Participants

This exploratory cross-sectional study was approved by the Botucatu Medical School Research Ethics Board (09871219.8.0000.5411). Written informed consent was obtained from all individuals before their inclusion in the study. All principles established in the Declaration of Helsinki were followed. As this study is an exploratory analysis aimed at generating new hypotheses, the sample size was estimated based on a previous study, and the minimum number to be included was 10 individuals in each group.

We evaluated Brazilian patients aged ≥ 18 years residing in the region of Botucatu, São Paulo, who were diagnosed with stage 5 CKD on ND and PD therapy for at least 3 months, from March to November 2019. The presence of stage 5 CKD was confirmed by a glomerular filtration rate (GFR) < 15 mL/min/ 1.73 m^2^ using the baseline creatinine value and the Chronic Kidney Disease Epidemiology Collaboration (CKD-EPI) equation. Patients were allocated to one of the two groups: ND (n = 10 patients with stage 5 CKD not on dialysis) and PD (n = 10 patients with CKD treated by PD). Groups were matched by age and body mass index (BMI).

At the time of patient enrollment, demographic and clinical information was recorded and blood samples were taken for plasma miRNA analysis and assessment of inflammatory biomarkers. Exclusion criteria were age over 80 years, active neoplasm and liver disease, acute infection, previous diagnosis of immunological diseases, kidney transplantation, pregnancy, limb amputation or physical limitation, use of pacemaker, and patients who did not agree to participate in the study.

### Biological Samples

Approximately 4 mL of blood was collected from each patient and transferred to tubes containing the anticoagulant EDTA. Plasma was separated from whole blood by centrifugation at 3,000 RPM for 15 minutes at 4°C. After this procedure, samples were identified, placed in polypropylene tubes, and stored at -80 ºC. Plasma was used for miRNA quantification upon RNA extraction and assessment of inflammatory biomarkers.

### Serum Pro-Inflammatory Biomarkers

The following inflammatory biomarkers were analyzed: interleukin 6 (IL-6), tumor necrosis factor-alpha (TNF-α), and high-sensitivity C-reactive protein (hs-CRP). IL-6 and TNF-α serum concentrations were assessed using the enzyme-linked immunosorbent assay (ELISA) (R&D System, Inc., Minneapolis, USA). hs-CRP was measured by turbidimetric immunoassay in an automatic analyzer system (Chemistry Analyzer BS-200, Mindray Medical International Limited, Shenzhen, China).

### RNA Extraction

RNA extraction was performed using 200 µL of plasma (7 patients per group) and the miRNeasy Serum/Plasma kit (Qiagen, São Paulo, SP, Brazil) following the manufacturer’s instructions. This method allows the purification of cell-free RNA needed for circulating miRNA quantification analysis. Briefly, RNA samples were first subjected to poly-A tail incorporation to 3´-end, followed by a second step of ligation and biotinylation of RNA 3´-poly A tail. Biotin-labeled RNA from each sample was hybridized to the GeneChip miRNA 4.0 array cartridge and detected with Avidin-Streptavidin-Phycoerythrin conjugate, which binds to biotin-labeled RNA with strong affinity. miRNA array cartridges were then placed into hybridization oven trays, which were loaded into the hybridization oven and incubated at 48˚C with 60 rpm rotation for 16 hours. Upon hybridization, each array was filled with an array-holding buffer and allowed to reach room temperature before washing and staining. The washing and staining steps were performed using the appropriate fluidics script for cartridge arrays on the fluidics station. Arrays were scanned and data was exported for further analysis using the Expression Console software (Affymetrix) for data summarization, normalization, and quality control.

### miRNA Expression Analysis using the GeneChip^®^ miRNA 4.0 Array (Affymetrix)

We used the Affymetrix GeneChip^®^ human miRNA 4.0 array (Thermo Fisher Scientific). This array contains 2,578 human mature miRNA probe sets and 2,025 human precursor (pre) miRNA probe sets. GeneChips were scanned using the Affymetrix GeneChip scanner G3000 7G with standard setting to capture signal intensities for the miRNAs. The raw intensity data were imported into Affymetrix Expression Console software (v1.4.1.46) for signal pre-processing including background correction utilizing the robust multi-array average (RMA) algorithm, median polish summarization from probe-to-probe set level of signal values, and the quantile method to normalize across multiple arrays. A detection call was recorded for each miRNA hybridization signal using the Affymetrix “Detection Above Background” (DABG) algorithm, which generates a p-value for signal above background. All steps followed the manufacturer’s protocol. This assay was performed at the Experimental Research Unit (UNIPEX), Botucatu Medical School, UNESP.

### Computational Data Analyses

Next, in order to map target genes regulated by the 9 identified miRNAs, we used the microRNA Integration Portal (miRDIP) tool (http://ophid.utoronto.ca/mirDIP/searchShared_OnMirs.jsp) applying the miRNA-gene matrix bioinformatics option^16^. After the identification of target genes regulated by miRNAs, we sought to identify whether genes were associated with biological pathways using the EnrichR tool (https://maayanlab.cloud/Enrichr/)^17^. We used String (https://string-db.org/) to generate the interaction network between miRNAs and target genes^18^. The network was visualized in Cytoscape (https://cytoscape.org/)^19^.

### Statistics

Data analyses were performed using SigmaPlot software for Windows v12.0 (Systat Software Inc., San Jose, CA, USA). Data are reported as mean ± SD, median (lower and upper quartiles), or percentage. For comparison between two groups, the Student’s t-test was used when continuous variables had a normal distribution and the Mann-Whitney test when they had a non-normal distribution. For categorical variables, the Chi-square test or Fisher’s exact test was used. Statistical significance was set as *p <* 0.05.

## Results

Twenty patients diagnosed with CKD were evaluated: 10 in the ND and 10 in the PD group. Patients had a mean age of 57.2 ± 11.8 years, 60% were males, and mean BMI was 28.8 ± 5.2 kg/m^2^. Among the etiologies of CKD, diabetic nephropathy was the most prevalent (35%), followed by undetermined cause (20%), glomerulopathies (10%), hypertensive nephropathy (10%), and other causes (25%).

Patients in the PD group had lower creatinine clearance and lower serum concentrations of urea, albumin, potassium, and HDL. In addition, they had higher serum creatinine and bicarbonate concentrations. Levels of hs-CRP [0.37 (0.07–1.40) vs. 3.90 (2.50–5.79), p = 0.038] and IL-6 [3.35 ± 3.08 vs. 6.82 ± 4.08, p = 0.046] were significantly lower in the ND group in comparison to the PD group, respectively. There were no differences between the groups regarding sex, age, and other laboratory data (Table 1).

**Table 1:** Demographic and laboratory data according to groups

Variables	Groups		p-value
	ND (n:10)	PD (n:10)	
Age, (y)^a^	58.9 ± 11.1	55.5 ± 12.7	0.532
Men, n (%)^b^	4 (40)	8 (80)	0.170
Cl Cr (mL/min/1.73 m^2^)^b^	11.4 (8.8–17.8)	4.3 (2.6–6.6)	<0.001
*Biochemistry*			
hs-CRP (mg/L)^b^	0.37 (0.07–1.40)	3.90 (2.50–5.79)	0.038
IL-6 (pg/mL)^a^	3.35 ± 3.08	6.82 ± 4.08	0.046
TNF-α (pg/mL)^a^	14.34 ± 3.56	15.45 ± 6.86	0.657
Urea (mg/dL)^a^	149.8 ± 29.2	108.5 ± 22.8	0.003
Creatinine (mg/dL)^b^	4.6 (3.0–5.0)	10.0 (7.0–13.5)	0.001
Blood glucose (mg/dL)^b^	95.0 (87.3–121.0)	97.5 (82.8–134.0)	0.929
Bicarbonate (mg/dL)^a^	22.4 ± 3.6	28.1 ± 3.4	0.003
Albumin (g/dL)^b^	4.2 (3.6–4.6)	3.6 (2.9–3.9)	0.019
Hemoglobin (g/dL)^a^	11.3 ± 1.6	12.2 ± 1.8	0.262
Calcium (mg/dL)^b^	9.0 (8.8–9.8)	9.1 (8.8–9.3)	0.902
Phosphorus (mg/dL)^b^	4.9 (4.5–5.6)	5.3 (5.0–7.2)	0.306
Potassium (mmol/L)^a^	4.9 ± 0.5	4.1 ± 0.5	0.005
PTH (pg/mL)^b^	229.0 (185.0–370.5)	192.0 (146.5–411.5)	0.401
Cholesterol (mg/dL)^a^	150.7 ± 25.1	141.9 ± 47.2	0.629
HDL (mg/dL)^a^	53.5 ± 14.5	34.7 ± 6.2	0.013
Triglycerides (mg/dL)^b^	117.0 (100.5–120.0)	110.0 (77.0–177.0)	0.930

Abbreviations – ND: Non-dialytic; PD: peritoneal dialysis; Cl Cr: clearance of creatinine; hsCRP: high sensitivity C-reactive protein; interleukin 6; TNF-α: tumor necrosis factor – alpha; PTH: parathyroid hormone; HDL: high-density lipoprotein. Notes – Data were expressed as mean ± SD, median (including lower and upper quartiles), or percentage.

^a^Student’s t.

^b^Mann-Whitney test.

^c^Chi-square test.

In relation to circulating miRNAs, 9 miRNAs were found to have a deregulated expression (FC ≥ 2 and p ≤ 0.05) in the PD group compared to the ND group: 8 were downregulated (miR-585-5p, miR-4793-3p, miR-1273g-3p, miR-6756-5p, miR 2861, miR-6727-5p, miR-3619-5p, and miR-122-5p) and 1 was upregulated (miR-3921) (Table 2).

**Table 2:** Deregulated miRNAs in PD group versus ND group (listed according to significant p-values)

miRNA	Fold change	p-value
miR-585-5p	−2.29	< 0.001
miR-4793-3p	−3.13	< 0.001
miR-1273g-3p	−5.68	0.002
miR-6756-5p	−2.22	0.002
miR-2861	−2.83	0.003
miR-6727-5p	−2.25	0.004
miR-3619-5p	−2.64	0.005
miR-122-5p	−4.93	0.001
miR-3921	2.79	< 0.001

**Figure 1:**
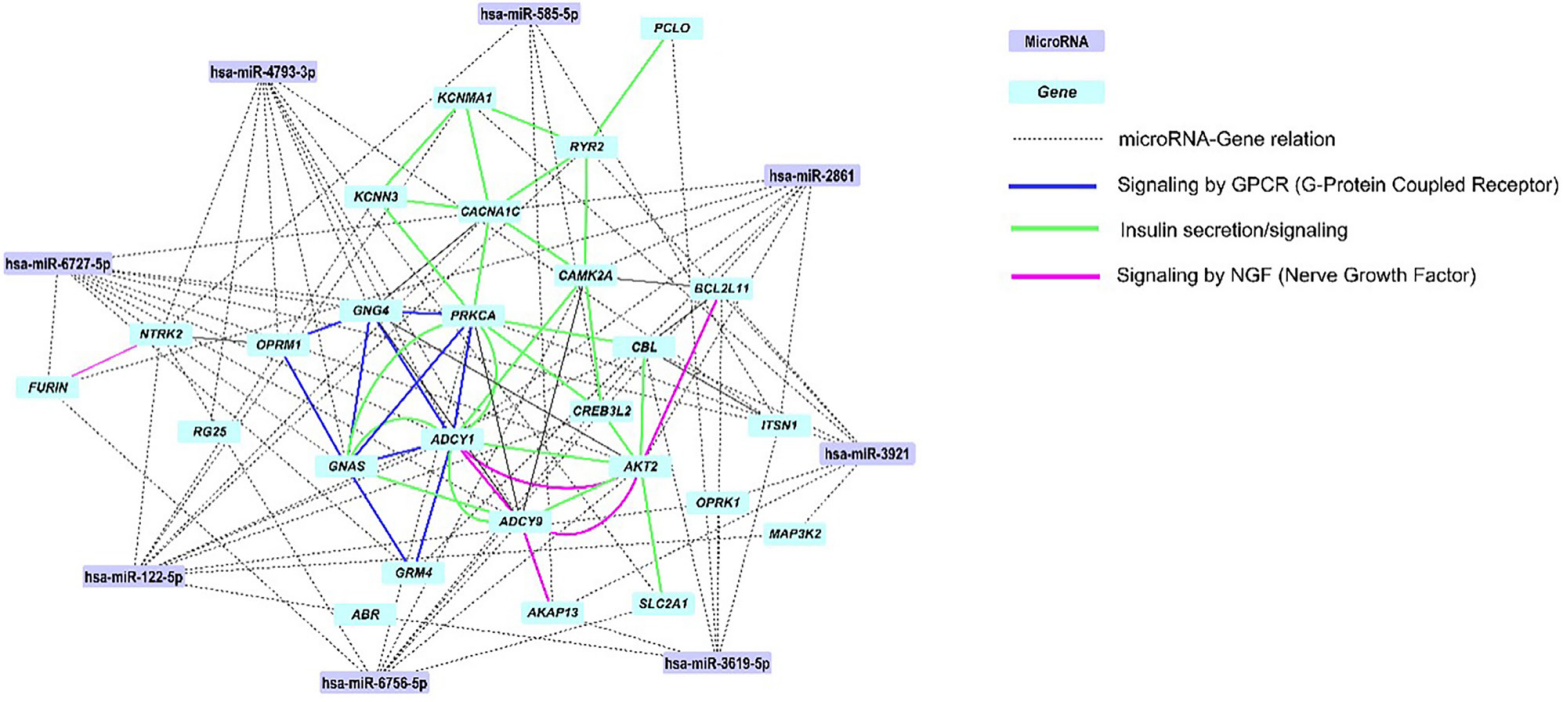
Network of miRNAs and target genes within pathways of Signaling by GPCR, Insulin secretion/signaling, and NGF signaling. The direct interactions between proteins encoded by target genes are represented by solid lines and highlighted in blue (GPCR signaling), green (Insulin secretion/signaling), and pink (NGF signaling). miRNAs regulate genes that interact in other pathways (interactions represented by dashed lines). (Created in: STRING and Cytoscape).

Results from miRDIP analysis showed 1,382 target genes regulated by all 9 miRNAs. We then performed pathway analysis and identified a subset of genes that are regulated by miRNAs and play roles in CKD pathogenesis (Table 3). Pathways were identified by three different database sources (KEGG 2021, WikiPathway 2021, and Reactome 2022), which increases the confidence of our findings. The network between miRNAs and target genes (Figure 1) highlights the regulatory pathways of G protein-coupled receptor (GPCR) signaling, insulin signaling, and nerve growth factor (NGF) signaling, which are relevant to CKD.

**Table 3:** Enriched pathways including miRNA target genes

Molecular pathways	Target genes	p value	Data bank
Signaling by GPCR and GPCR downstream signaling	CHRM3, EDN2, ITPR2, OPRK1, ECE1, PRKCA, ADCY1, OPRM1, RGS4, RGS5, GRM4, GNG4, GNB1, GNAS, RGS8, PRKACB	1.142e-23	Reactome 2022
Insulin secretion/signaling	CHRM3, RYR2, SLC2A1, ATP1A4, PRKCA, ADCY1, ATP1A1, CACNA1C, PCLO, CREB3L2, KCNMA1, GNAS, KCNN3, PRKACB MAP3K2, AKT2, CBL, ADCY9, CAMK2A, ADCY1	2.119e-29	KEGG 2021
Signaling by NGF	ABR, AKAP13, NTRK2, ADCY9, BCL2L11, AKT2, ITSN1, FURIN, ADCY1	9.922e-15	Reactome 2022
ECM-receptor interaction	LAMB3, ITGA2, LAMC2, NPNT, HSPG2, THBS3, SV2C, TNN, SV2A, ITGA10, DAG1, TNR, AGRN, CD44	0.0027	KEGG 2021

## Discussion

This study evaluated the circulating miRNAs levels and proinflammatory biomarkers in patients with end-stage CKD subjected to different treatment modalities. We showed that 9 circulating miRNAs were deregulated in PD patients compared to ND treatment, and these miRNAs are predicted to regulate (with very high levels of confidence), a subset of 36 genes within pathways that are relevant to CKD pathogenesis in PD. The GPCR signaling, insulin secretion/resistance, NGF signaling, metabolism, and other pathways identified are linked to fibrosis and inflammation.

Interestingly, miRNAs were found to regulate genes within the GPCR pathway, which contributes to the understanding of molecular physiology and the identification of novel biomarkers potentially useful for improved therapeutics in CKD. GPCR signaling emerged as a central mechanism potentially modulated by the deregulated miRNAs in PD patients. GPCRs play crucial roles in renal physiology, including regulation of blood flow, glomerular filtration, and tubular reabsorption processes^20^. Notably, *GNAS*, a gene encoding the stimulatory G-protein alpha subunit, was predicted to be a high-confidence target. *GNAS* is essential for parathyroid hormone (PTH) signaling, which promotes phosphate reabsorption in the proximal tubule and contributes to calcium-phosphate homeostasis—a process often disrupted in CKD and particularly relevant in dialysis patients^16^. Another relevant target is *PRKCA* (protein kinase C alpha), which plays a key role in the regulation of angiogenesis, inflammation, and glomerular permeability^21^. The dysregulation of *PRKCA* has been implicated in diabetic nephropathy and renal fibrosis, further supporting the pathological relevance of our findings. These specific gene-function associations help to contextualize the potential impact of miRNA-mediated post-transcriptional regulation in the pathogenesis of CKD in PD patients.

Muralidharan et al.^22^ evaluated miRNA expression in body fluids (plasma and urine) from patients with glomerular filtration rate (eGFR) ≥ 30 vs. < 30 mL·min-^1^·1.73 m-^2^. These authors showed 384 urinary and 266 plasma miRNAs differentially expressed between the two groups of patients. According to sample type and groups, several miRNAs were up- or downregulated. Further pathway analysis showed that most miRNAs were associated with TGFβ, which plays a role in kidney fibrosis^22^. In our study, we also showed circulating miRNAs associated with genes regulating extracellular matrix (ECM)-receptor interactions. Increased ECM accumulation is known to contribute to cellular organization disruption and is characteristic of fibrosis associated with end-stage CKD^23,24^. The understanding and identification of ECM proteins involved in CKD may be useful for the development of non-invasive biomarkers and novel treatment approaches^25,26^.

Regarding inflammatory biomarkers, we found that patients undergoing PD had more intense inflammation compared to ND patients. In general, ND-CKD patients are expected to exhibit a lower pro-inflammatory state due to the clearance of cytokines via residual renal function. In contrast, individuals undergoing PD tend to exhibit a more pronounced pro-inflammatory profile, driven by increased insulin resistance, hyperglycemia, weight, dyslipidemia, risks of peritonitis, and catheter-related infections. This inflammatory process is associated with increased catabolism and insulin resistance. The potential role of insulin in the kidney is evidenced by the association of CKD with type 2 diabetes and non-alcoholic fatty liver disease^27^. Here, we identified miRNA target genes implicated in mechanisms of insulin signaling/resistance in PD patients. Insulin resistance is highly prevalent in CKD and is associated with a complex interplay of alterations including protein-energy wasting and systemic chronic inflammation^28^. Nerve Growth Factor signaling, which has been identified as one of the significant pathways regulated by miRNAs and target genes, was implicated in unpaired kidney function, more specifically in glomerular response to tissue injury^29^.

A previous report showed that the type of adipose tissue (white or brown) differentially influences the expression of circulating miRNAs involved with metabolism regulation^15^. Adipose tissue is an important source of exosomal-derived miRNAs, including miR-221, miR-201, miR-222, miR-16, miR-325, miR-743-b, and miR-98^15^. Even though there are differences between miRNAs expressed in exosomes and plasma^30^, these differences appear to be small^31^. A study of healthy individuals found that the expression of miRNAs appears similar in plasma and exosome^29^, likely reflecting a state of equilibrium between circulating plasma and exosomes. Validating these findings in CKD patients undergoing different types of treatment remains a target for future studies. However, little is known about the influence of miRNAs on body mass in healthy individuals or patients with CKD. Therefore, here we assessed body composition to contribute to the interpretation of our results. Patients with CKD without dialysis therapy can present a reduction in both adiposity and muscle mass, which could be directly related to anorexia, a sedentary lifestyle, a low protein diet, ageing, and hypercatabolism due to the accumulation of uremic toxins. On the other hand, individuals undergoing PD treatment tend to have a pro-inflammatory profile, caused by a higher risk of peritonitis, catheter infection, insulin resistance, hyperglycemia, and adipogenesis^32^.

In our study, both groups had excess body fat and were not at risk for protein energetic wasting (PEW) or sarcopenia. We must highlight the considerable accumulation of body fat, even in patients who did not receive glucose infusion into the peritoneal cavity (ND patients). Despite this finding, we did not find a statistically significant difference in fat and muscle mass between groups, which suggests that body composition did not influence the expression of miRNAs and pro-inflammatory cytokines in our patients. Although our patients did not have considerable muscle depletion, a very common condition among individuals with CKD, some patients were obese. Also, it is worth noting that the patients had good clinical control and nutritional status. Therefore, our results should not be generalized to all patients with CKD.

The main scientific challenge of this study was to perform a broad analysis of miRNAs, inflammatory biomarkers, and body composition in patients with different stages of CKD. As strengths of the study, we highlight the use of DXA and multifrequency BIA, which are widely recommended methods for assessing body composition, including individuals with CKD undergoing dialysis treatment^5^. The assessment of muscle mass (including quantity and functionality) and adipose tissue using these methods minimized bias and strengthened our findings. Our study has some limitations, including a small sample size from a single medical center. Nonetheless, our findings are novel and contribute to the identification of circulating miRNAs associated with end-stage CKD in PD patients. The identified miRNAs are potent regulators of target genes within biological networks important to CKD, thus providing a scientific basis for further validation using independent cohorts.

In conclusion, patients on PD treatment showed deregulated circulating levels of a subset of miRNAs and higher serum concentrations of hs-CRP and IL-6 in comparison to the ND patients. Genes regulated by miRNAs are mainly associated with GPCR signaling, insulin resistance and energy metabolism, playing roles in fibrosis and inflammatory-associated functions. Our results contribute to the identification of circulating miRNAs and pro-inflammatory mediators that are likely influenced by PD treatment.

## Data Availability

The datasets generated and/or analyzed during the current study are available from the corresponding author upon reasonable request.

## References

[1] Jager KJ, Kovesdy C, Langham R, Rosenberg M, Jha V, Zoccali C (2019). A single number for advocacy and communication-worldwide more than 850 million individuals have kidney diseases.. Kidney Int.

[2] Prasad N, Patel MR, Chandra A, Rangaswamy D, Sinha A, Bhadauria D (2017). Measured glomerular filtration rate at dialysis initiation and clinical outcomes of Indian peritoneal dialysis patients.. Indian J Nephrol.

[3] Kramer A, Pippias M, Noordzij M, Stel VS, Afentakis N, Ambühl PM (2018). The European Renal Association – European Dialysis and Transplant Association (ERA-EDTA) registry annual report 2015: a summary.. Clin Kidney J.

[4] Ribeiro HS, Neri SGR, Oliveira JS, Bennett PN, Viana JL, Lima RM (2022). Association between sarcopenia and clinical outcomes in chronic kidney disease patients: a systematic review and meta-analysis.. Clin Nutr Edinb Scotl..

[5] Ikizler TA, Burrowes JD, Byham-Gray LD, Campbell KL, Carrero JJ, Chan W (2020). KDOQI clinical practice guideline for nutrition in CKD: 2020 update.. Am J Kidney Dis.

[6] Ziolkowski SL, Long J, Baker JF, Chertow GM, Leonard MB (2019). Relative sarcopenia and mortality and the modifying effects of chronic kidney disease and adiposity.. J Cachexia Sarcopenia Muscle.

[7] Wang AYM, Sea MMM, Ho ZSY, Lui SF, Li PKT, Woo J (2005). Evaluation of handgrip strength as a nutritional marker and prognostic indicator in peritoneal dialysis patients.. Am J Clin Nutr.

[8] Trionfini P, Benigni A, Remuzzi G (2015). MicroRNAs in kidney physiology and disease.. Nat Rev Nephrol.

[9] Romaine SPR, Tomaszewski M, Condorelli G, Samani NJ (2015). MicroRNAs in cardiovascular disease: an introduction for clinicians.. Heart.

[10] Friedman RC, Farh KKH, Burge CB, Bartel DP (2009). Most mammalian mRNAs are conserved targets of microRNAs.. Genome Res.

[11] Wei Q, Mi QS, Dong Z (2013). The regulation and function of microRNAs in kidney diseases.. IUBMB Life.

[12] Fan J, Kou X, Yang Y, Chen N (2016). MicroRNA-regulated proinflammatory cytokines in sarcopenia.. Mediators Inflamm.

[13] Contreras J, Rao DS (2012). MicroRNAs in inflammation and immune responses.. Leukemia.

[14] Arner P, Kulyté A (2015). MicroRNA regulatory networks in human adipose tissue and obesity.. Nat Rev Endocrinol.

[15] Thomou T, Mori MA, Dreyfuss JM, Konishi M, Sakaguchi M, Wolfrum C (2017). Adipose-derived circulating miRNAs regulate gene expression in other tissues.. Nature.

[16] Qin L, Lin Y, Li Q (2022). The role of GNAS in parathyroid hormone signaling and phosphate homeostasis in chronic kidney disease.. Am J Physiol Renal Physiol.

[17] Tokar T, Pastrello C, Rossos AEM, Abovsky M, Hauschild AC, Tsay M (2018). mirDIP 4.1-integrative database of human microRNA target predictions.. Nucleic Acids Res.

[18] Szklarczyk D, Kirsch R, Koutrouli M, Nastou K, Mehryary F, Hachilif R (2023). The STRING database in 2023: protein–protein association networks and functional enrichment analyses for any sequenced genome of interest.. Nucleic Acids Res.

[19] Shannon P, Markiel A, Ozier O, Baliga NS, Wang JT, Ramage D (2003). Cytoscape: a software environment for integrated models of biomolecular interaction networks.. Genome Res.

[20] Ghosh S, Sengupta A, Sharma S, Biswas T (2021). G protein-coupled receptors and their emerging role in kidney diseases.. Front Physiol.

[21] Lee K, Kim J, Yoon J (2020). Protein kinase C-alpha modulates inflammatory signaling pathways in renal fibrosis.. Kidney Int Rep.

[22] Muralidharan J, Ramezani A, Hubal M, Knoblach S, Shrivastav S, Karandish S (2017). Extracellular microRNA signature in chronic kidney disease.. Am J Physiol Renal Physiol.

[23] Rayego-Mateos S, Campillo S, Rodrigues-Diez RR, Tejera-Muñoz A, Marquez-Exposito L, Goldschmeding R (2021). Interplay between extracellular matrix components and cellular and molecular mechanisms in kidney fibrosis.. Clin Sci.

[24] Kim KP, Williams CE, Lemmon CA (2022). Cell-matrix interactions in renal fibrosis.. Kidney Dial..

[25] Genovese F, Manresa AA, Leeming DJ, Karsdal MA, Boor P (2014). The extracellular matrix in the kidney: a source of novel non-invasive biomarkers of kidney fibrosis?. Fibrogenesis Tissue Repair.

[26] Bülow RD, Boor P (2019). Extracellular matrix in kidney fibrosis: more than just a scaffold.. J Histochem Cytochem Off J Histochem Soc..

[27] Pina AF, Borges DO, Meneses MJ, Branco P, Birne R, Vilasi A (2020). Insulin: trigger and target of renal functions.. Front Cell Dev Biol.

[28] Liao MT, Sung CC, Hung KC, Wu CC, Lo L, Lu KC (2012). Insulin resistance in patients with chronic kidney disease.. J Biomed Biotechnol.

[29] Bonofiglio R, Antonucci MT, Papalia T, Romeo F, Capocasale G, Caroleo MC (2007). Nerve growth factor (NGF) and NGF receptor expression in diseased human kidneys.. J Nephrol.

[30] Wu W, Wu X, Cheng Z, Yang Z, Lu M, Cheng J (2022). Differentially expressed microRNAs in peritoneal dialysis effluent-derived exosomes from the patients with ultrafiltration failure.. Genet Res.

[31] Tian F, Shen Y, Chen Z, Li R, Ge Q (2017). No significant difference between plasma miRNAs and plasma-derived exosomal miRNAs from healthy people.. BioMed Res Int.

[32] Kim JK, Kim YS, Song YR, Kim HJ, Kim SG, Moon SJ (2015). Excessive weight gain during the first year of peritoneal dialysis is associated with inflammation, diabetes mellitus, and a rapid decrease in residual renal function.. PLoS One.

