# Supplementary material for: Peritoneal dialysis influences microRNA expression and pro-inflammatory response: results from a cross-sectional study
Source: J Bras Nefrol. 2025 Dec 19;48(1):e20240218. doi: 10.1590/2175-8239-JBN-2024-0218en (PMC12820807; doi:10.1590/2175-8239-JBN-2024-0218en)
Supplement: Translation

# Translation: A diálise peritoneal exerce influência na expressão de microRNAS e na resposta pró-inflamatória: resultados de um estudo transversal

**DOI:** 10.1590/2175-8239-JBN-2024-0218pt

**Authors:** Nara Aline Costa, Amanda Gomes Pereira, Hellen Christina Neves Rodrigues, Lilian Cuppari, Tainara Francini Felix, Iael Weissberg Minutentag, Patricia Pintor Reis, André Luis Balbi, Bertha Furlan Polegato, Paula Schmidt Azevedo, Leonardo Antonio Mamede Zornoff, Sérgio Alberto Rupp de Paiva, Daniela Ponce, Marcelo Macedo Rogero, Marcos Ferreira Minicucci

**Affiliations:** 1 Universidade Federal de Goiás, Faculdade de Nutrição, Goiânia, GO, Brasil.; 2 Universidade Estadual Paulista, Faculdade de Medicina, Departamento de Medicina Interna, Botucatu, SP, Brasil.; 3 Universidade Federal de São Paulo, Disciplina de Nefrologia, São Paulo, SP, Brasil.; 4 Universidade Estadual Paulista, Faculdade de Medicina, Unidade de Pesquisa Experimental, Botucatu, SP, Brasil.; 5 Universidade Estadual Paulista, Faculdade de Medicina, Departamento de Cirurgia e Ortopedia, Botucatu, SP, Brasil.; 6 Universidade de São Paulo, Faculdade de Saúde Pública, Departamento de Nutrição, São Paulo, SP, Brasil.

**Keywords:** Insuficiência Renal Crônica, MicroRNAs, Inflamação, Redes e Vias Metabólicas

## Abstract

**Introdução:**

A investigação de microRNAs (miRNAs) circulantes e da resposta inflamatória associada aos diferentes estágios da DRC pode revelar biomarcadores da patogênese da doença. Nosso objetivo foi identificar diferenças na expressão de miRNAs circulantes entre pacientes em diálise peritoneal (DP) e não dialíticos (ND), determinando redes regulatórias da interação miRNA-gene-alvo e vias potencialmente envolvidas na patogênese da doença.

**Métodos:**

Estudo transversal exploratório incluindo pacientes ND e em DP com DRC estágio 5 e idade superior a 18 anos. Avaliaram-se biomarcadores inflamatórios e perfis de expressão de miRNA circulantes.

**Resultados:**

Foram incluídos 20 pacientes (57,2 ± 11,8 anos). Níveis de proteína C-reativa de alta sensibilidade [0,37 (0,07–1,40) vs. 3,90 (2,50–5,79); p = 0,038] e interleucina-6 [3,35 ± 3,08 vs. 6,82 ± 4,08; p = 0,046] foram significativamente menores no grupo ND comparados ao grupo DP, respectivamente. Nove miRNAs apresentaram desregulação significativa (*fold change* (FC) ≥ 2 e p ≤ 0,05) no grupo DP comparado ao grupo ND. Análises computacionais revelaram grande número de genes-alvo comumente regulados por pelo menos dois dos miRNAs identificados. A análise de enriquecimento de vias demonstrou que sinalização por receptor acoplado à proteína G (GPCR), secreção/resistência à insulina e metabolismo energético estiveram entre as vias significativas reguladas pelos genes-alvo do miRNA.

**Conclusões:**

Pacientes em tratamento com DP apresentaram níveis circulantes desregulados dos 9 miRNAs identificados e biomarcadores inflamatórios séricos mais elevados, comparados aos pacientes ND. Genes regulados pelos miRNAs associam-se principalmente à sinalização por GPCR, resistência à insulina e metabolismo energético, desempenhando papéis na fibrose e funções associadas à inflamação.

## Introdução

A doença renal crônica (DRC) nos estágios 1 a 5 afeta mais de 10% da população mundial, o que equivale a aproximadamente 843,6 milhões de indivíduos. O ônus da DRC representa um desafio significativo para a saúde global, especialmente em países de baixa e média renda, uma vez que se tornou uma das principais causas de mortalidade, destacando a necessidade urgente de esforços aprimorados de prevenção e tratamento^1^. A progressão para a DRC em estágio terminal requer tratamento com terapias renais substitutivas, incluindo diálise peritoneal (DP). Pacientes em tratamento não dialítico (ND) geralmente apresentam um estado pró-inflamatório de baixo grau devido à preservação da função renal residual, o que permite a depuração de citocinas inflamatórias. Em contrapartida, indivíduos em DP frequentemente apresentam aumento da inflamação, atribuído a fatores como resistência à insulina, hiperglicemia, ganho de peso, peritonite e infecções relacionadas ao cateter^2,3^. Além disso, sabe-se que o tecido adiposo e a maior intensidade da inflamação contribuem para o aumento da morbidade e mortalidade, especialmente entre pacientes em terapia de diálise^4–7^. Essas diferenças ressaltam a importância de retardar a progressão da DRC para os estágios dialíticos, uma vez que a manutenção da função renal é crucial para mitigar a inflamação e a progressão da doença.

Nessa perspectiva, novas linhas de pesquisa têm sugerido que os microRNAs (miRNAs), associados a características sistêmicas como calcificação vascular, aterosclerose e distúrbios minerais e ósseos, desempenham um papel no desenvolvimento e progressão da DRC^8^. Os miRNAs são pequenos RNAs não codificantes com um papel regulatório pós-transcricional na expressão gênica^9,10^. Foram desenvolvidas estratégias minimamente invasivas para quantificar os níveis de miRNA em fluidos corporais - como o sangue - de pacientes com diversas condições clínicas. A análise dos miRNAs plasmáticos oferece inúmeras vantagens, uma vez que essas moléculas são abundantes nos fluidos corporais, apresentam elevada estabilidade e são de fácil detecção^11^. É importante destacar que os miRNAs regulam genes-alvo envolvidos em importantes processos biológicos, incluindo a fibrose em resposta a lesões teciduais e danos renais. Além disso, os miRNAs, incluindo miR-155, miR-146a e miR-21, têm sido implicados na regulação da resposta inflamatória e, se alterados, podem contribuir para a perda intensa de massa muscular^12,13^.

Estudos sugerem que existe uma relação complexa e integrada entre os miRNAs, as respostas imune e inflamatória e a composição corporal; porém, os dados publicados são insuficientes para a compreensão completa dessa relação na DRC^14,15^. Até onde temos conhecimento, nenhum estudo avaliou os níveis de miRNAs circulantes em pacientes não dialíticos (ND) e em DP. Nosso objetivo foi identificar diferenças na expressão de miRNAs circulantes entre pacientes em DP e ND, bem como determinar as redes regulatórias de genes-alvo de miRNAs e as vias potencialmente envolvidas na patogênese da doença.

## Métodos

### Desenho do Estudo e Participantes

Este estudo transversal exploratório foi aprovado pelo Comitê de Ética em Pesquisa da Faculdade de Medicina de Botucatu (09871219.8.0000.5411). O consentimento livre e esclarecido por escrito foi obtido de todos os indivíduos antes de sua inclusão no estudo. Todos os princípios estabelecidos na Declaração de Helsinque foram seguidos. Por se tratar de uma análise exploratória com o objetivo de gerar novas hipóteses, o tamanho da amostra foi estimado com base em um estudo prévio, sendo definido um número mínimo de 10 indivíduos em cada grupo.

Foram avaliados pacientes brasileiros com idade ≥ 18 anos residentes na região de Botucatu, São Paulo, diagnosticados com DRC estágio 5 em tratamento ND e em DP por pelo menos 3 meses, de março a novembro de 2019. A presença de DRC estágio 5 foi confirmada por uma taxa de filtração glomerular (TFG) < 15 mL/min/1,73 m^2^ utilizando o valor basal da creatinina e a equação Chronic Kidney Disease Epidemiology Collaboration (CKD-EPI). Os pacientes foram distribuídos em dois grupos: ND (n = 10 pacientes com DRC estágio 5 não submetidos à diálise) e DP (n = 10 pacientes com DRC tratados por DP). Os grupos foram pareados por idade e índice de massa corporal (IMC).

No momento da inclusão dos pacientes, foram registradas informações demográficas e clínicas, e amostras de sangue foram coletadas para análise de miRNA plasmático e avaliação de biomarcadores inflamatórios. Os critérios de exclusão foram idade superior a 80 anos, neoplasia ativa e doença hepática, infecção aguda, diagnóstico prévio de doenças imunológicas, transplante renal, gestação, amputação de membro ou limitação física, uso de marcapasso e pacientes que não concordaram em participar do estudo.

### Amostras Biológicas

Aproximadamente 4 ml de sangue foram coletados de cada paciente e transferidos para tubos contendo o anticoagulante EDTA. O plasma foi separado do sangue total por centrifugação a 3.000 RPM por 15 minutos a 4 °C. Após este procedimento, as amostras foram identificadas, acondicionadas em tubos de polipropileno e armazenadas a -80 ºC. O plasma foi utilizado para quantificação de miRNA mediante extração de RNA e avaliação de biomarcadores inflamatórios.

### Biomarcadores Pró-Inflamatórios Séricos

Foram analisados os seguintes biomarcadores inflamatórios: interleucina 6 (IL-6), fator de necrose tumoral alfa (TNF-α>) e proteína C-reativa de alta sensibilidade (hs-CRP). As concentrações séricas de IL-6 e TNF-α> foram avaliadas por meio do ensaio imunoenzimático (ELISA) (R&D System, Inc., Minneapolis, EUA). A hs-CRP foi mensurada por imunoensaio turbidimétrico em um sistema analisador automático (Chemistry Analyzer BS-200, Mindray Medical International Limited, Shenzhen, China).

### Extração de RNA

A extração do RNA foi realizada utilizando 200 µL de plasma (7 pacientes por grupo) e o kit miRNeasy Serum/Plasma (Qiagen, São Paulo, SP, Brasil), seguindo as instruções do fabricante. Este método permite a purificação do RNA livre de células necessário para a análise de quantificação do miRNA circulante. Resumidamente, as amostras de RNA foram inicialmente submetidas à incorporação de cauda poli-A na extremidade 3’, seguida por uma segunda etapa de ligação e biotinilação da cauda poli-A de 3’ do RNA. O RNA marcado com biotina de cada amostra foi hibridizado no cartucho GeneChip miRNA 4.0 array e detectado com o conjugado Avidina-Estreptavidina-Ficoeritrina, que se liga ao RNA biotinilado com forte afinidade. Os cartuchos de de miRNA array foram então colocados em bandejas de forno de hibridização, as quais foram carregadas no forno de hibridização e incubadas a 48 °C com rotação de 60 rpm durante 16 horas. Após a hibridização, cada array foi preenchido com uma solução tampão (array-holding buffer) e deixou-se atingir a temperatura ambiente antes da lavagem e coloração. As etapas de lavagem e coloração foram realizadas utilizando o script fluídico apropriado para cartuchos de array na estação fluídica. Os arrays foram digitalizados e os dados foram exportados para análise subsequente com o software Expression Console (Affymetrix) para sumarização, normalização e controle de qualidade dos dados.

### Análise da Expressão de miRNA Utilizando o *GeneChip*® miRNA 4.0 Array (Affymetrix)

Utilizamos o GeneChip® human miRNA 4.0 array da Affymetrix (Thermo Fisher Scientific). Este array contém 2.578 conjuntos de sondas de miRNA humano maduro e 2.025 conjuntos de sondas de miRNA humano precursor (pré-miRNA). Os GeneChips foram digitalizados utilizando o scanner Affymetrix GeneChip G3000 7G com configuração padrão para capturar as intensidades de sinal dos miRNAs. Os dados brutos de intensidade foram importados para o software Affymetrix Expression Console (v1.4.1.46) para pré-processamento de sinais, incluindo correção de fundo utilizando o algoritmo RMA (robust multi-array average), sumarização por median polish a partir do nível de valores de sinal do conjunto de sondas e método quantílico para normalização entre múltiplos arrays. Foi registrada uma detecção para cada sinal de hibridização de miRNA utilizando o algoritmo “Detection Above Background” (DABG) da Affymetrix, que gera um valor p para sinais acima do ruído de fundo. Todas as etapas seguiram o protocolo do fabricante. Este ensaio foi realizado na Unidade de Pesquisa Experimental (UNIPEX) da Faculdade de Medicina de Botucatu, UNESP.

### Análises de Dados Computacionais

Em seguida, com o objetivo de mapear os genes-alvo regulados pelos 9 miRNAs identificados, utilizamos a ferramenta microRNA Integration Portal (miRDIP) (http://ophid.utoronto.ca/mirDIP/searchShared_OnMirs.jsp) aplicando a opção de bioinformática de matriz miRNA-gene^16^. Após a identificação dos genes-alvo regulados pelos miRNAs, buscamos identificar se os genes estavam associados a vias biológicas utilizando a ferramenta EnrichR (https://maayanlab.cloud/Enrichr/)^17^. Utilizamos o String (https://string-db.org/) para gerar a rede de interação entre os miRNAs e os genes-alvo^18^. A rede foi visualizada no Cytoscape (https://cytoscape.org/)^19^.

### Estatísticas

As análises dos dados foram realizadas utilizando o software SigmaPlot para Windows v12.0 (Systat Software Inc., San Jose, CA, EUA). Os dados são apresentados como média + DP, mediana (quartis inferior e superior) ou porcentagem. Para comparação entre dois grupos, utilizou-se o teste t de Student quando as variáveis contínuas apresentaram distribuição normal e o teste de Mann-Whitney quando apresentaram distribuição não normal. Para variáveis categóricas, aplicou-se o teste do qui-quadrado ou o teste exato de Fisher. O nível de significância estatística foi definido como p < 0,05.

## Resultados

Foram avaliados 20 pacientes diagnosticados com DRC: 10 no grupo ND e 10 no grupo DP. A média de idade dos pacientes foi de 57,2 ± 11,8 anos, 60% eram do sexo masculino e o IMC médio foi de 28,8 ± 5,2 kg/m^2^. Entre as etiologias da DRC, a nefropatia diabética foi a mais prevalente (35%), seguida por causa indeterminada (20%), glomerulopatias (10%), nefropatia hipertensiva (10%) e outras causas (25%).

Os pacientes do grupo DP apresentaram menor depuração da creatinina e menores concentrações séricas de ureia, albumina, potássio e HDL. Além disso, apresentaram maiores concentrações séricas de creatinina e bicarbonato. Os níveis de hs-CRP [0,37 (0,07–1,40) vs. 3,90 (2,50–5,79; p = 0,038] e IL-6 [3,35 + 3,08 vs. 6,82 + 4,08; p = 0,046] foram significativamente mais baixos no grupo ND em comparação ao grupo DP, respectivamente. Não houve diferenças entre os grupos em relação ao sexo, idade e demais dados laboratoriais (Tabela 1).

**Tabela 1:** Dados demográficos e laboratoriais de acordo com os grupos

Variáveis	Grupos		Valor de p
	ND (n:10)	PD (n:10)	
Idade, (a)^a^	58,9 ± 11,1	55,5 ± 12,7	0,532
Sexo masculino, n (%)^c^	4 (40)	8 (80)	0,170
Cl Cr (mL/min/1,73 m^2^)^b^	11,4 (8,8–17,8)	4,3 (2,6–6,6)	<0,001
*Bioquímica*			
hs-CRP (mg/L)^b^	0,37 (0,07–1,40)	3,90 (2,50–5,79)	0,038
IL-6 (pg/mL)^a^	3,35 ± 3,08	6,82 ± 4,08	0,046
TNF-α> (pg/mL)^a^	14,34 ± 3,56	15,45 ± 6,86	0,657
Ureia (mg/dL)^a^	149,8 ± 29,2	108,5 ± 22,8	0,003
Creatinina (mg/dL)^b^	4,6 (3,0–5,0)	10,0 (7,0–13,5)	0,001
Glicemia (mg/dL)^b^	95,0 (87,3–121,0)	97,5 (82,8–134,0)	0,929
Bicarbonato (mg/dL)^a^	22,4 ± 3,6	28,1 ± 3,4	0,003
Albumina (g/dL)^b^	4,2 (3,6–4,6)	3,6 (2,9–3,9)	0,019
Hemoglobina (g/dL)^a^	11,3 ± 1,6	12,2 ± 1,8	0,262
Cálcio (mg/dL)^b^	9,0 (8,8–9,8)	9,1 (8,8–9,3)	0,902
Fósforo (mg/dL)^b^	4,9 (4,5–5,6)	5,3 (5,0–7,2)	0,306
Potássio (mmol/L)^a^	4,9 ± 0,5	4,1 ± 0,5	0,005
PTH (pg/mL)^b^	229,0 (185,0–370,5)	192,0 (146,5–411,5)	0,401
Colesterol (mg/dL)^a^	150,7 ± 25,1	141,9 ± 47,2	0,629
HDL (mg/dL)^a^	53,5 ± 14,5	34,7 ± 6,2	0,013
Triglicérides (mg/dL)^b^	117,0 (100,5–120,0)	110,0 (77,0–177,0)	0,930

Abreviaturas – ND: Não dialítico; DP: diálise peritoneal; Cl Cr: depuração de creatinina; hsCRP: proteína C-reativa de alta sensibilidade; interleucina 6; TNF-α: fator de necrose tumoral alfa; PTH: paratormônio; HDL: lipoproteína de alta densidade. Notas – Os dados foram expressos como média + DP, mediana (incluindo quartis inferior e superior) ou porcentagem.

^a^Teste t de Student.

^b^Teste de Mann-Whitney.

^c^Teste qui-quadrado.

Em relação aos miRNAs circulantes, 9 miRNAs demonstraram expressão desregulada (FC ≥ 2 e p ≤ 0,05) no grupo DP em comparação ao grupo ND: 8 estavam subexpressos (miR-585-5p, miR-4793-3p, miR-1273g-3p, miR-6756-5p, miR 2861, miR-6727-5p, miR-3619-5p e miR-122-5p) e 1 estava superexpresso (miR-3921) (Tabela 2).

**Tabela 2:** miRNAs desregulados no grupo DP versus grupo ND (listados de acordo com valores de p significativos)

miRNA	Fold change	Valor de p
miR-585-5p	−2,29	<0,001
miR-4793-3p	−3,13	<0,001
miR-1273g-3p	−5,68	0,002
miR-6756-5p	−2,22	0,002
miR-2861	−2,83	0,003
miR-6727-5p	−2,25	0,004
miR-3619-5p	−2,64	0,005
miR-122-5p	−4,93	0,001
miR-3921	2,79	<0,001

Os resultados da análise pelo miRDIP revelaram 1.382 genes-alvo regulados por todos os 9 miRNAs. Em seguida, realizamos uma análise de vias e identificamos um subconjunto de genes que são regulados por miRNAs e desempenham papéis na patogênese da DRC (Tabela 3). As vias foram identificadas por três fontes de banco de dados diferentes (KEGG 2021, WikiPathway 2021 e Reactome 2022), o que aumenta a confiabilidade de nossos achados. A rede entre os miRNAs e os genes-alvo (Figura 1) evidencia as vias regulatórias de sinalização do receptor acoplado à proteína G (GPCR), sinalização da insulina e sinalização do fator de crescimento nervoso (NGF), que são relevantes para a DRC.

**Tabela 3:** Vias enriquecidas, incluindo genes-alvo de miRNA

Vias Moleculares	Genes-alvo	Valor de p	Banco de dados
Sinalização por GPCR e sinalização a jusante do GPCR	CHRM3, EDN2, ITPR2, OPRK1, ECE1, PRKCA, ADCY1, OPRM1, RGS4, RGS5, GRM4, GNG4, GNB1, GNAS, RGS8, PRKACB	1,142e-23	*Reactome* 2022
Secreção/sinalização da insulina	CHRM3, RYR2, SLC2A1, ATP1A4, PRKCA, ADCY1, ATP1A1, CACNA1C, PCLO, CREB3L2, KCNMA1, GNAS, KCNN3, PRKACB, MAP3K2, AKT2, CBL, ADCY9, CAMK2A, ADCY1	2,119e-29	KEGG 2021
Sinalização por NGF	ABR, AKAP13, NTRK2, ADCY9, BCL2L11, AKT2, ITSN1, FURIN, ADCY1	9,922e-15	*Reactom*e 2022
Interação MEC-receptor	LAMB3, ITGA2, LAMC2, NPNT, HSPG2, THBS3, SV2C, TNN, SV2A, ITGA10, DAG1, TNR, AGRN, CD44	0,0027	KEGG 2021

**Figura 1:** Rede de miRNAs e genes-alvo nas vias de sinalização por GPCR, secreção/sinalização de insulina e sinalização por NGF. As interações diretas entre proteínas codificadas por genes-alvo são representadas por linhas contínuas e destacadas em azul (sinalização por GPCR), verde (secreção/sinalização de insulina) e rosa (sinalização por NGF). Os miRNAs regulam genes que interagem em outras vias (interações representadas por linhas tracejadas). (Criado em: STRING e Cytoscape).

## Discussão

Este estudo avaliou os níveis de miRNAs circulantes e biomarcadores pró-inflamatórios em pacientes com DRC em estágio terminal submetidos a diferentes modalidades de tratamento. Demonstramos que nove miRNAs circulantes estavam desregulados em pacientes em DP, em comparação ao tratamento ND, e prevê-se que esses miRNAs regulem (com níveis de confiança bastante elevados) um subconjunto de 36 genes dentro de vias relevantes para a patogênese da DRC na DP. A sinalização GPCR, a secreção/resistência à insulina, a sinalização NGF, o metabolismo e outras vias identificadas estão associadas à fibrose e à inflamação.

Curiosamente, constatou-se que os miRNAs regulam genes na via GPCR, o que contribui para a compreensão da fisiologia molecular e identificação de novos biomarcadores potencialmente úteis para o aprimoramento de terapêuticas na DRC. A sinalização GPCR emergiu como um mecanismo central potencialmente modulado por miRNAs desregulados em pacientes submetidos à DP. Os GPCRs desempenham papéis cruciais na fisiologia renal, incluindo regulação do fluxo sanguíneo, filtração glomerular e processos de reabsorção tubular^20^. Notavelmente, o GNAS, um gene que codifica a subunidade alfa da proteína G estimulatória, foi previsto como um alvo de alta confiabilidade. O GNAS é essencial para a sinalização do paratormônio (PTH), que promove a reabsorção de fosfato no túbulo proximal e contribui para a homeostase do cálcio-fosfato — um processo frequentemente interrompido na DRC e particularmente relevante em pacientes em diálise^16^. Outro alvo relevante é a PRKCA (proteína quinase C alfa), que desempenha um papel crucial na regulação da angiogênese, inflamação e permeabilidade glomerular^21^. A desregulação da PRKCA tem sido implicada na nefropatia diabética e na fibrose renal, reforçando ainda mais a relevância patológica de nossos achados. Essas associações específicas entre genes e funções ajudam a contextualizar o impacto potencial da regulação pós-transcricional mediada por miRNA na patogênese da DRC em pacientes em DP.

Muralidharan et al.^22^ avaliaram a expressão do miRNA em fluidos corporais (plasma e urina) de pacientes com taxa de filtração glomerular (TFGe) ≥ 30 vs. < 30 mL*·*min-^1^ *·*1,73 m-^2^. Esses autores demonstraram 384 miRNAs urinários e 266 plasmáticos expressos de forma diferenciada entre os dois grupos de pacientes. De acordo com o tipo de amostra e os grupos, diversos miRNAs apresentaram regulação positiva ou negativa. Análises adicionais de vias demonstraram que a maioria dos miRNAs estava associada ao TGFβ>, que desempenha papel importante na fibrose renal^22^. Em nosso estudo, também demonstramos a presença de miRNAs circulantes associados a genes que regulam as interações entre matriz extracelular (MEC) e receptores. Sabe-se que o aumento do acúmulo de MEC contribui para a ruptura da organização celular e é característico de fibrose associada à DRC em estágio terminal^23,24^. A compreensão e identificação das proteínas da MEC envolvidas na DRC podem ser úteis para o desenvolvimento de biomarcadores não invasivos e novas abordagens terapêuticas^25,26^.

Em relação aos biomarcadores inflamatórios, observamos que pacientes submetidos à DP apresentaram inflamação mais intensa em comparação aos pacientes ND. De modo geral, espera-se que os pacientes com DRC-ND exibam um estado pró-inflamatório de baixo grau devido à depuração de citocinas pela função renal residual. Em contrapartida, os indivíduos em DP tendem a apresentar um perfil pró-inflamatório mais pronunciado, impulsionado pelo aumento da resistência à insulina, hiperglicemia, peso, dislipidemia, riscos de peritonite e infecções relacionadas ao cateter. Este processo inflamatório está associado ao aumento do catabolismo e à resistência à insulina. O papel potencial da insulina no rim é evidenciado pela associação da DRC com diabetes tipo 2 e doença hepática gordurosa não alcoólica^27^. Neste estudo, identificamos genes-alvo de miRNA implicados nos mecanismos de sinalização/resistência à insulina em pacientes em DP. A resistência à insulina é altamente prevalente na DRC e está associada a uma interação complexa de alterações, incluindo desperdício de energia proteica e inflamação crônica sistêmica^28^. A sinalização do fator de crescimento nervoso, identificada como uma das vias significativas reguladas por miRNAs e genes-alvo, foi implicada na função renal prejudicada, mais especificamente na resposta glomerular à lesão tecidual^29^.

Um relato prévio demonstrou que o tipo de tecido adiposo (branco ou marrom) influencia de maneira diferenciada a expressão de miRNAs circulantes envolvidos na regulação do metabolismo^15^. O tecido adiposo representa uma importante fonte de miRNAs derivados de exossomos, incluindo miR-221, miR-201, miR-222, miR-16, miR-325, miR-743-b e miR-98^15^. Embora existam diferenças entre os miRNAs expressos nos exossomos e no plasma^30^, essas diferenças parecem ser pouco significativas^31^. Um estudo realizado em indivíduos saudáveis constatou que a expressão de miRNAs parece semelhante no plasma e no exossomo^29^, refletindo provavelmente um estado de equilíbrio entre o plasma circulante e os exossomos. A validação destes achados em pacientes com DRC submetidos a diferentes tipos de tratamento permanece como objetivo para estudos futuros. No entanto, pouco se sabe sobre a influência dos miRNAs na massa corporal em indivíduos saudáveis ou pacientes com DRC. Portanto, no presente estudo avaliamos a composição corporal a fim de contribuir para a interpretação de nossos resultados. Pacientes com DRC sem terapia dialítica podem apresentar redução tanto da adiposidade quanto da massa muscular, o que pode estar diretamente relacionado à anorexia, ao estilo de vida sedentário, à dieta hipoproteica, ao envelhecimento e ao hipercatabolismo devido ao acúmulo de toxinas urêmicas. Por outro lado, indivíduos em tratamento com DP tendem a apresentar um perfil pró-inflamatório, decorrente do maior risco de peritonite, infecção do cateter, resistência à insulina, hiperglicemia e adipogênese^32^.

Em nosso estudo, ambos os grupos apresentaram excesso de gordura corporal e não estavam em risco de desperdício de energia proteica (DEP) ou sarcopenia. Deve-se destacar o acúmulo considerável de gordura corporal, mesmo em pacientes que não receberam infusão de glicose na cavidade peritoneal (pacientes ND). Apesar desse achado, não observamos diferença estatisticamente significativa na massa gorda e muscular entre os grupos, o que sugere que a composição corporal não influenciou a expressão de miRNAs e citocinas pró-inflamatórias em nossos pacientes. Embora nossos pacientes não apresentassem depleção de massa muscular considerável – uma condição bastante comum entre indivíduos com DRC –, alguns eram obesos. Além disso, é importante ressaltar que os pacientes apresentavam bom controle clínico e estado nutricional. Portanto, nossos resultados não devem ser generalizados para todos os pacientes com DRC.

O principal desafio científico deste estudo consistiu em realizar uma análise abrangente de miRNAs, biomarcadores inflamatórios e composição corporal em pacientes com diferentes estágios de DRC. Como pontos fortes do estudo, destacamos o uso da DXA e da BIA multifrequencial, métodos amplamente recomendados para a avaliação da composição corporal, incluindo indivíduos com DRC submetidos a tratamento dialítico^5^. A avaliação da massa muscular (incluindo quantidade e funcionalidade) e do tecido adiposo utilizando estes métodos minimizou vieses e fortaleceu nossos achados. Nosso estudo apresenta algumas limitações, incluindo o pequeno tamanho da amostra proveniente de um único centro médico. No entanto, nossos achados são inéditos e contribuem para a identificação de miRNAs circulantes associados à DRC em estágio terminal em pacientes em DP. Os miRNAs identificados são potentes reguladores de genes-alvo em redes biológicas importantes para a DRC, fornecendo, assim, uma base científica para validação adicional utilizando coortes independentes.

Em conclusão, os pacientes em tratamento com DP apresentaram níveis circulantes desregulados de um subconjunto de miRNAs e concentrações séricas mais elevadas de hs-CRP e IL-6 em comparação aos pacientes ND. Os genes regulados por miRNAs estão principalmente associados à sinalização GPCR, resistência à insulina e metabolismo energético, desempenhando papéis na fibrose e em funções associadas à inflamação. Nossos resultados contribuem para a identificação de miRNAs circulantes e mediadores pró-inflamatórios que provavelmente são influenciados pelo tratamento com DP.

## Data Availability

Os conjuntos de dados gerados e/ou analisados durante o presente estudo estão disponíveis mediante solicitação razoável ao autor correspondente.
